# P03-019 - Anakinra for sweet syndrome treatment

**DOI:** 10.1186/1546-0096-11-S1-A217

**Published:** 2013-11-08

**Authors:** G Passaro, L Cerrito, M Giovinale, A Marinaro, A Soriano, D Rigante, R Manna

**Affiliations:** 1 Periodic Fever Research Center, Catholic University of Sacred Heart, Rome, Italy

## Introduction

Sweet syndrome (SS) is a rare autoinflamamtory neutrophilic dermatosis, sometimes associated with hematologic malignancies. We describe two cases of SS related to hematologic diseases successfully treated with an interleukin-1 (IL-1) receptor antagonist (Anakinra).

## Case report

**1-** A 72-year-old man with diagnosis of Relapsing Polychondritis and Myelodysplastic syndrome (MDS) was referred for a 4-yr history of recurrent fever (37.5 to 39°C) accompanied by sudden onset of cutaneous purple papules on the extremities and hypodermic painful nodules on the forehead and scalp. The patient suffered of autoimmune atrophic gastritis and severe metasteroidal osteoporosis, but also complained of recurrent auricular chondritis, recurrent conjunctival inflammation, hearing loss, aphonia, and itching. Blood tests revealed high C-reactive protein (43.2 mg/l), pancytopenia, 1/160 titer of ANA, and elevated beta2microglobulin (10.10 mg/ml). A host of blood tests and broncho-alveolar lavage fluid were negative. Bone marrow (BM) biopsy confirmed MDS. Histopatological evaluation of papules demonstrated neutrophilic nodular dermatitis with karyorrhexis without vasculitis, consistent with SS.

**2-** A 43-year-old man, with a 3-yr history of fever, periodic bone pain, and presence of maculo-papular erythematous skin lesions came to our observation. Histological examination of skin lesions demonstrated a diffuse infiltrate, consisting predominantly of mature neutrophils located in the upper dermis, framed as SS. MRI of pelvis and spine showed multiple sclerotic vertebral bone lesions. BM biopsy did not detect tumor cells, but instead identified inflammatory cells with intense sclerosis and calcification. Diagnosis of Chronic Recurrent Multifocal Osteomyellitis (CRMO) was established. Treatment with etoricoxib, colchicine, corticosteroid was only partially successful, whereas bisphosphonate and anti-TNF therapy were completely uneffective.

In both these patients Anakinra was administered (100 mg/day by daily subcutaneous injection), obtaining the suppression of neutrophylic-mediated dermatologic manifestations. In the first case we also observed fever spike reduction, whereas in the second one there was the complete remission of fever and bone pain.

## Discussion

Pathogenesis of SS involves cytokines and chemokines, as granulocyte-macrophage colony-stimulating factor and interleukins (e.g, IL-1, IL-3, IL-6, and IL-8). The optimal effect of Anakinra in these cases supports the major contributing role of IL-1beta in the physio-pathogenetic process of SS. The choice of alternative strategies, as anti-IL1beta therapy, is feasible in the absence of detectable infections to reduce adverse effects of long-term steroid therapy or in cases of insufficient response to conventional treatment.

## Competing interests

None Declared.
